# The Willoughby Medical College*

**Published:** 1837

**Authors:** 


### Art. V.—The Willoughby Medical College.*

In a former number of this journal, we made mention of the new institution, of which a further account is contained in the pamphlets we have just quoted.

Forty years ago, the celebrated French traveller, Volney, after visiting the West, wrote down in his travels, that of the whole, he would "*prefer to live, a hundred years hence, on the margin of Lake Erie.*" At that time, we believe there was no settlement on the lake shore west of Erie, in the State of Pennsylvania. The progress of society and improvement, has been such as to show, that the French philosopher looked at this country with a prophetic eye. Towns and institutions have already sprung up on the shores of the lake and the little rivers which descend from the summit level of our State, as if by *spontaneous generation*. Among them are the village and University of Willoughby.

The catalogue of officers and students which now lies before us, presents the names of five medical professors—Drs. Ackley, Trowbridge, Peixotto, Cassels, and Smith, (every school must have a Smith,) and a class of 51 pupils, which, for its locality and infancy, may be regarded as promising. The fees amount to $60. The length of session and the requirements for graduation, are substantially the same as in the other schools of the United States. A capacious and well-appointed edifice has been erected, and nothing but perseverance would seem to be necessary to success.

The introductory lecture of Professor Peixotto, is a respectable production; abounding in just, and, to his pupils, exciting reflections. His theme is the institution, with a peroration on several ancient and modern luminaries of the profession, including his preceptor, the late distinguished Dr. David Hosack, of New York.

As he passes on, he makes the following quaint but appropriate observation on empyricism:—

"I know too well from past experience, that no positive law can

---

* Catalogue (No. 4.) of the Officers, Professors and Students, of the Willoughby University of Lake Erie, in Willoughby, Cuyahoga county, Ohio, 1836–7.

Introductory Lecture delivered at the same, at the opening of the session of 1836–7, by Daniel L. M. Peixotto, M. D., Professor of the Theory and Practice, &c.

reach or grasp the slippery eels of quackery. They will glide from under the touch, and continue to pollute the earth with their venom and slime. But if they cannot be directly crushed by state enactments, a healthful public opinion may disarm them and hurl them into the hiding places whence they should never have been allowed to emerge."

In referring to difficulties with which the trustees of the institution, while in its formiug state, had been compelled to grapple, he makes the following remarks, which are applicable to other institutions than his own:—

"Over the early history of the institution, the vexatious disappointments of the Trustees, and their subsequent failure for a time, it is most prudent to cast a veil. Few indeed are the undertakings of man which are not destined, more especially at their outset, to encounter obstacles, and to be threatened with utter failure. An indomitable spirit bent on public good, can and will overcome all resistance. Insidious foes lurking and prowling about with honey on their lips, but gall and wormwood in their hearts, may for a while raise their hydra heads, and pour forth their vituperative denunciations, but the light of the cloudless sun does not shed its vivifying rays with more certainty to adorn and enrich this globe, than truth must and shall prevail over foul mouthed slander and impotent malignity. The clouds, that erst darkened over the horizon of this institution, are dispelled,—and the evil-eyed prophets, who assumed the gratuitous responsibility of asserting that this very edifice, in which we are now assembled, would never be erected, have now the mortification, so painful to themselves, so gratifying to the friends of science, to see its ample accommodations, scarcely surpassed in any section of the Union, thrown open to the public view and use."

We extract another sentence touching a different matter:—

"*Ex cathedra* assertions may be listened to with respectful silence in the lecture room, but in the retirement of his closet, the student will, over his books, analyze all he has heard, and separate, with intuitive precision, gratuitous declamation from sound reasoning, and exaggerated assertions from simple natural facts."

It would be pleasant to believe, that students of medicine are accustomed thus to analyze and depurate what is funnelled into their ears, by boisterous and declamatory teachers; but we fear that very few of them practice that kind of chemistry. The *ex cathedra* falls with authority upon too many young minds. Our students should not be trusted to in this matter. It is for the guardians of our schools to interpose their power, and preserve the *cathedræ medicæ* from superficial declaimers, as well as dull and drowsy systematists.

On a former occasion, we have expressed the opinion, that the lake country in the West should have a medical institution; but added, that Cleveland, from its greater size, would seem to be a better site than Willoughby. We shall not, however, pursue this idea, but, leaving it with our friends in that portion of the State, conclude this hasty notice, with our best wishes for their success, and the assurance that as long as they labor to raise their school to a high and still higher level, they will find us ready to "lend a hand," however feeble, to its elevation. D.

